# *Proneotermes macondianus*, a new drywood termite from Colombia and expanded distribution of *Proneotermes* in the Neotropics (Isoptera, Kalotermitidae)

**DOI:** 10.3897/zookeys.623.9677

**Published:** 2016-10-11

**Authors:** Robin Casalla, Rudolf H. Scheffrahn, Judith Korb

**Affiliations:** 1 Universität Freiburg. Evolutionary Biology & Ecology. Hauptstrasse 1, Freiburg 79104 Germany; 2 Universidad del Norte. Departamento de Química y Biología. Kilómetro 5 Antigua vía Puerto Colombia, Barranquilla Colombia; 3 University of Florida. Fort Lauderdale Research & Education Center, 3205 College Avenue Davie, Florida 33314 United States

**Keywords:** Northern Colombian coast, Proneotermes keys, Proneotermes macondianus sp. n., tropical dry forest

## Abstract

After more than one hundred years, a new drywood termite of the genus *Proneotermes* is described from the tropical dry forest in the Caribbean coast of Colombia. Morphological and genetic analyses are given for *Proneotermes macondianus* **sp. n**. This termite occurs in tropical dry forests in small colonies inside thin branches of dry wood. The soldier of *Proneotermes macondianus* is smaller and the genal horns are angled outward compared to the other two described *Proneotermes* species. The imago wings are unusually short and wide. Genetic analyses for COII, 12S, and 16S genes show less than three percent difference between sample localities of *Proneotermes macondianus*. Intergeneric comparison with selected kalotermitid genera indicates that *Bifiditermes* is the most closely related genus of those sequenced. New morphological descriptions and morphometric measurements of *Proneotermes latifrons* based on the soldier caste are also included. Neotropical locality records for *Proneotermes latifrons and Proneotermes perezi* are provided.

## Introduction

Colombia’s diverse ecoregions harbour high termite diversity (Morrone 2006, Robledo et al. 2014). Vargas-Niño et al. (2005) and Krishna et al. (2013) together report 29 genera of higher termites (Termitidae). Ten additional generic termitid records, mostly Apicotermitinae, have been collected in Colombia (Scheffrahn unpubl. data). Among the Kalotermitidae, six genera (*Calcaritermes*, *Comatermes*, *Cryptotermes*, *Glyptotermes*, *Incisitermes*, and *Neotermes*) are known from Colombia (Krishna et al. 2013, Rodríguez et al. 2012). The tropical dry forest of Colombia’s Caribbean coast has recently revealed a new species and two new records of *Cryptotermes* (Casalla et al. 2016).

For more than a century, the genus *Proneotermes* was represented by two species, *Proneotermes latifrons* (Silvestri, 1901) from Venezuela and *Proneotermes perezi* (Holmgren 1911) from Costa Rica (Krishna et al. 2013). DNA barcoding is a molecular tool used to identify and to track the evolutionary biology of species (Thompson et al. 2000, Inward et al. 2007, Hausberger et al. 2011, Bourguignon et al. 2014). Evolutionary analyses within the Kalotermitidae are incomplete and limited to some genera (Legendre et al. 2008). Hence a comparative genetic analysis can help to determine relationships for *Proneotermes*.

In this paper, a new species of *Proneotermes* is described, *Proneotermes macondianus*. In addition, new morphological descriptions are included for the soldier of *Proneotermes latifrons* and new soldier measurements provided for *Proneotermes perezi* as well as new locality records for *Proneotermes* in the Neotropics.

## Methods

### Study sites and sampling

**Figure 1.**
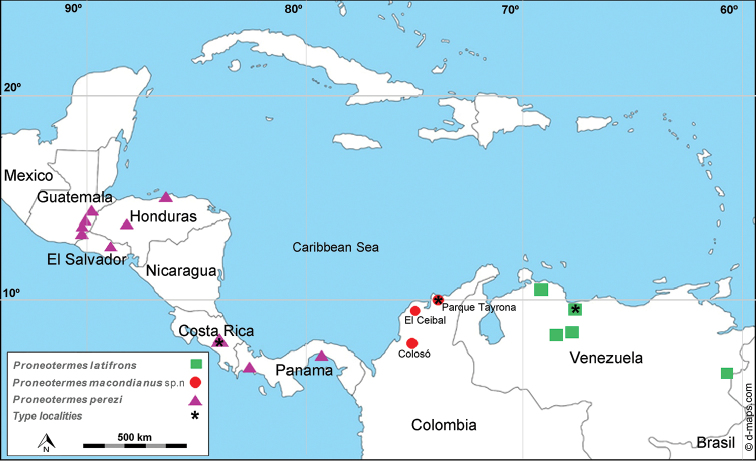
Sampling localities for *Proneotermes macondianus* sp. n in Colombia and new records from University of Florida collection for *Proneotermes latifrons* and *Proneotermes perezi*.

Three study sites in a tropical dry forest near Colombia’s Caribbean coast were selected and surveyed during July 2014 and August 2015 (Fig. 1). The area of “Los Primates” in the mountains of municipality of Colosó, Sucre and the “El Ceibal” in Santa Catalina Bolívar, are part of the system of protected areas, while the “El Parque Tayrona” is a Natural National Park of Colombia in Santa Marta, Magdalena. These forests are one of the best preserved areas of tropical dry forest in the Colombian Caribbean coast (Instituto de Investigación Alexander von Humboldt 2014). Samples of a new *Proneotermes* were collected in those places using a standardized sampling protocol (Jones and Eggleton 2000, Hausberger and Korb 2015) that included collecting small dry branches and dry wood on the ground. Specimens were preserved in 100% ethanol for DNA analysis and 80% ethanol for museum curation.

### Identification

Morphometrics for *Proneotermes latifrons* and *Proneotermes perezi* were obtained from specimens from the University of Florida Termite Collection, Davie, Florida. Specimens of *Proneotermes macondianus* sp. n. were also sequenced for genetic comparisons. Total DNA was extracted from pseudergates and alate imagoes heads using the CTAB protocol (Doyle and Doyle 1987). PCRs and sequencing were performed for mitochondrial gene fragments from cytochrome oxidase II (COII) (~740 bp), 12S rDNA (~385 bp), and 16S rDNA (~480 bp) as described in Hausberger et al. (2011).

For the three different haplotypes of *Proneotermes macondianus* from the northern Colombian coast (separated ~200 km from each other), we used the combined COII, 12S, and 16S nucleotide sequences to calculate the *p*-distance (3000^th^ Bootstrap replications, Gamma Distributed and Transitions + Transversions).

Due to limited availability of mitochondrial gene sequences for Kalotermitidae in National Center for Biotechnology Information (NCBI), we restricted our phylogenetic analysis to only the COII fragment. Twelve genera of Kalotermitidae and *Cryptocercus punctulatus* as the outgroup were used (Table 1). Sequences were aligned with MUSCLE alignment algorithm as implemented in MEGA 7.0 with default settings (Kumar et al. 2016). A phylogenetic tree was inferred based in a Bayesian approach using MrBayes 3.2.1. (Ronquist and Huelsenbeck and 2003) (10^7^ generations with every 1000^th^ tree sampled, using the default of four chains). After checking for convergence, we discarded 50% as burn-in. The resultant tree was visualized using FigTree 1.4.2 (http://tree.bio.ed.ac.uk/software/figtree/). Additionally, MEGA 7.0 was also employed (Kumar et al. 2016) to calculate *p*-distances (as described above) between all species using the COII fragment. All positions containing gaps and missing data were eliminated.

**Table 1.** GenBank accession numbers for COII, 12S and 16S sequences.

Species	Accession COII	GenBank Accession 12S	Accession 16S
*Cryptocercus punctulatus*	DQ007637.1	–	–
*Bifiditermes improbus*	AF189079.1	–	–
*Bifiditermes improbus*	AF189080.1	–	–
*Calcaritermes temnocephalus*	EU253877.1	–	–
*Comatermes perfectus*	EU253878.1	–	–
*Cryptotermes cavifrons*	FN377810.1	–	–
*Cryptotermes longicollis*	FN377806.1	–	–
*Epicalotermes mkuzii*	DQ442125.1	–	–
*Glyptotermes brevicornis*	AF189096.1	–	–
*Glyptotermes iridipenis*	AF189096.2	–	–
*Glyptotermes satsumensis*	KP026257.1	–	–
*Incisitermes immigrans*	AB109542.1	–	–
*Incisitermes tabogae*	EU253880.1	–	–
*Kalotermes flavicollis*	DQ442147.1	–	–
*Marginitermes* sp.	KJ907844.1	–	–
*Neotermes castaneus*	HQ215844.1	–	–
*Neotermes holmgreni*	EU253882.1	–	–
*Neotermes insularis*	AF189105.1	–	–
*Postelectrotermes amplus*	DQ442147.1	–	–
*Postelectrotermes howa*	EU253883.1	–	–
*Procryptotermes leewardensis*	EU253884.1	–	–
Proneotermes macondianusCE*	**KX267096**	**KX267094**	**KX267091**
Proneotermes macondianusCO**	**KX267097**	**KX267093**	**KX267090**
Proneotermes macondianusPT***	**KX267098**	**KX267095**	**KX267092**

Samples localites: CE* = El Ceibal (Santa Catalina, Bolívar) , CO** = Los Primates (Colosó, Sucre) , PT*** = Parque Tayrona (Santa Marta, Magdalena)

### Imaging and measurements

Specimens were suspended in Hand Sanitizer and images were taken with a Leica MC205 C stereomicroscope coupled to a Leica MC190 HD digital camera. The software Helicon Focus was used to stack pictures. Measurements were done following Roonwal (1969). Wings and mandibles were detached and mounted onto slides and edited with Photoshop CS5 V12.0.

### Deposit

Voucher specimens are held at the University of Freiburg, Germany. The holotype soldier and paratypes of *Proneotermes macondianus* will be deposited at the Natural History Museum of the Alexander von Humboldt Institute of Bogotá (MIAvH) and a paratype soldier at the collection of the American Museum of Natural History, New York. De-alates (wings detached) and pseudergates of *Proneotermes macondianus* will be part of the collection of the Department of Chemistry and Biology at the Universidad del Norte, Barranquilla, Colombia.

## Results

### Family Kalotermitidae Froggatt, 1897 Genus *Proneotermes* Holmgren, 1911

#### Proneotermes macondianus sp. n.

Taxon classificationAnimaliaIsopteraKalotermitidae

http://zoobank.org/AB0F7282-534A-448D-AC80-86E697A18E9E

##### Diagnosis.

The *Proneotermes macondianus* soldier is smaller and the head capsule lighter than those of *Proneotermes latifrons* and *Proneotermes perezi*. In *Proneotermes macondianus*, the lateral margins of the genal horns angle outward from the sides of the head capsule while, in the other two species, the lateral margins of the genal horns remain in line with the head capsule. The mandibular humps of *Proneotermes macondianus* are more pronounced and rounded than in *Proneotermes latifrons* and *Proneotermes perezi*. Both *Proneotermes latifrons* and *Proneotermes perezi* have more robust rugosity on the frons than *Proneotermes macondianus*. The imago of *Proneotermes macondianus* is smaller and has much shorter, wider, darker, and more punctate wings than that of *Proneotermes perezi*.

##### Description.

**Figure 2.**
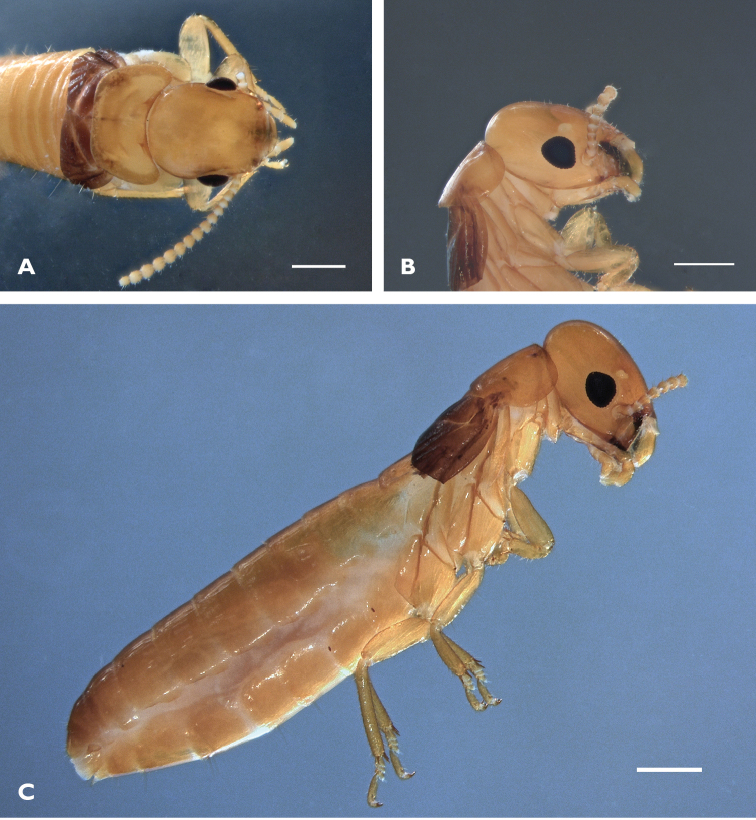
*Proneotermes macondianus* sp. n. imago: **A** Head in dorsal **B** lateral, and **C** whole body in lateral. Scale bars: 0.5 mm.

**Figure 3.**
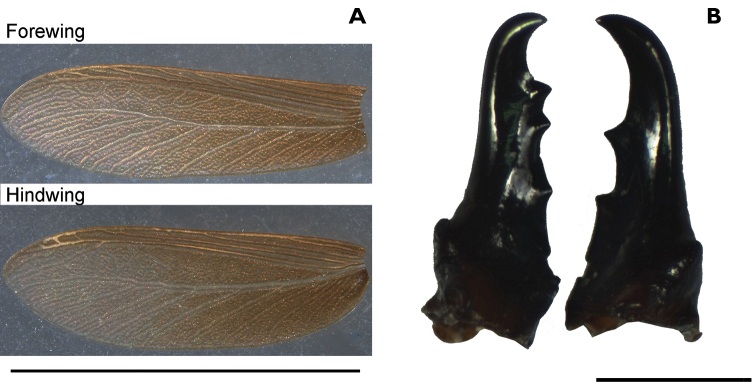
*Proneotermes macondianus* sp. n. Alate: **A** fore- and hindwing, scale bar: 5 mm. Soldier: **B** mandibles, scale bar: 1 mm.

Imago (Figs 2, 3A, Table 2). Head dorsal view: yellowish weakly trapezoidal, eyes moderately protruding and small, diameter 0.30 mm (Fig. 2A–B). Ocellus oval and almost touching eye (Fig. 2B). Antenna with 15 articles. Pronotum broader than head (Fig. 2A). Forewing with all major veins running parallel; subcosta running from suture to costal margin about 1/5 length of wing, radius to 1/3 wing length, radial sector with 4-6 branches, media less sclerotized than anterior veins, and cubitus unsclerotized Wings brownish, especially near scale suture, membrane nodular; unusually wide and relatively short. Fore wing with a very long suture line margin; scale much darker that body pigmentation (Fig. 3A). Measurements are reported in Table 2.

**Table 2.** Morphometrical measurements for *Proneotermes macondianus* sp. n. imagoes.

No.	Measurements in mm (n = 10).	Mean	SD	Range
1	Head length with labrum	1.28	0.06	1.18–1.35
2	Head length to postclypeus	1.14	0.04	1.06–1.19
3	Head width, maximum at eyes	0.95	0.04	0.88–1.01
4	Eye diameter, maximum	0.30	0.02	0.26–0.31
5	Eye to head base, minimum	0.14	0.01	0.12–0.16
6	Ocellus diameter	0.11	0.02	0.09–0.13
7	Pronotum maximum width	1.05	0.05	0.94–1.13
8	Pronotum maximum length	0.66	0.02	0.63–0.69
9	Total length without wings	5.45	0.33	5.04–5.87
10	Total length with wings (n = 1)	7.52	-	-
11	Fore wing length to suture (n = 1)	5.24	-	-
12	Fore wing maximum width (n = 1)	1.63	-	-
13	No. antennal articulations	9	2.4	7–15

**Figure 4.**
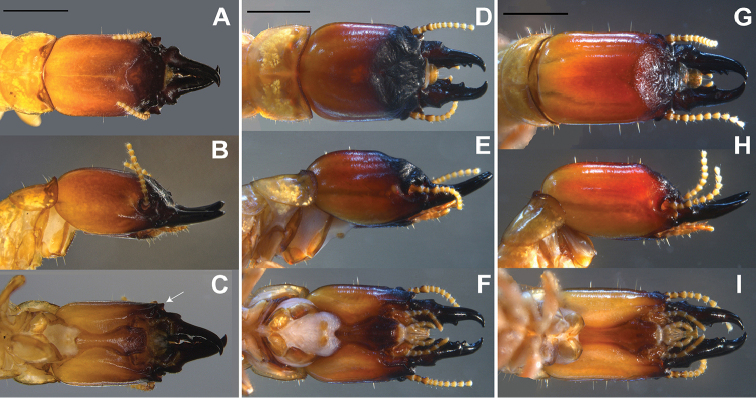
Soldier heads of *Proneotermes macondianus* sp. n. (**A–C**), *Proneotermes latifrons* (**D–F**), *Proneotermes perezi* (**G–I**). Head in dorsal (**A, D, G**), lateral (**B, E, H**) and ventral position (**C, F, I**). Arrow in 4C denote genal horn projected prominently in *Proneotermes macondianus* sp. n. Scale bars: 1 mm.

**Soldier** (Figs 3B, 4, Table 3). Head in dorsal view with postclypeus almost black, grading to ferruginous orange near frontal flange, and yellow at occiput (Fig. 4A). Dorsal view with head elongate and sides parallel, frons wide and shallow and faint frontal flange (Fig. 4B). Eye spots distinct, unpigmented. Mandibles completely black (Fig. 3B, 4A–C). Pronotum yellowish with anterior borders brown. Frons angles below vertex approx. 43°. Rugosity vestigial on the frons or vertex regions of the head. Frontal horns robust and project towards the front (Fig. 4A). Genal horns prominent in dorsal view, angled antero-laterally about 45°. Mandible tips bend about 60-65° from longitudinal axis of mandibular blade, prominent dentition, with rounded and pronounced mandibular humps: left hump larger than right (Figs 3B, 4A). Postmentum somewhat constricted in middle, as cup-shaped (Fig. 4C). Third antennal article enlarged and sclerotized, formula 2<3>4=5=6 and 11 articles. Pronotum as broad as head; anterior emarginate. Measurements are reported in Table 3. The soldiers from the Tayrona National Park (Santa Marta, Magdalena) showed slightly darker coloration than those from the samples sites at El Ceibal (Santa Catalina, Bolívar) and Colosó (Colosó, Sucre).

**Table 3.** Morphometrical measurements for *Proneotermes macondianus* sp. n. soldiers.

No.	Measurements in mm (n = 11).	Mean	SD	Range
1	Head length to tip of mandibles	2.75	0.11	2.50–2.95
2	Head length to frontal horns	1.72	0.10	1.54–1.90
3	Frontal flange width	1.03	0.04	0.92–1.08
4	Genal horns outside span	1.12	0.05	1.02–1.19
5	Head width max.	1.23	0.06	1.14–1.32
6	Head height excluding postmentum	0.99	0.06	0.85–1.08
7	Pronotum max. width	1.19	0.07	1.05–1.27
8	Pronotum max. length	0.82	0.05	0.73–0.87
9	Left mandible length, tip to ventral condyle (n = 1)	1.10	-	-
10	Total length	6.57	0.44	5.43–6.98
11	No. antennal articulations	11	0.7	10–12

##### Genetic analysis.

**Figure 5.**
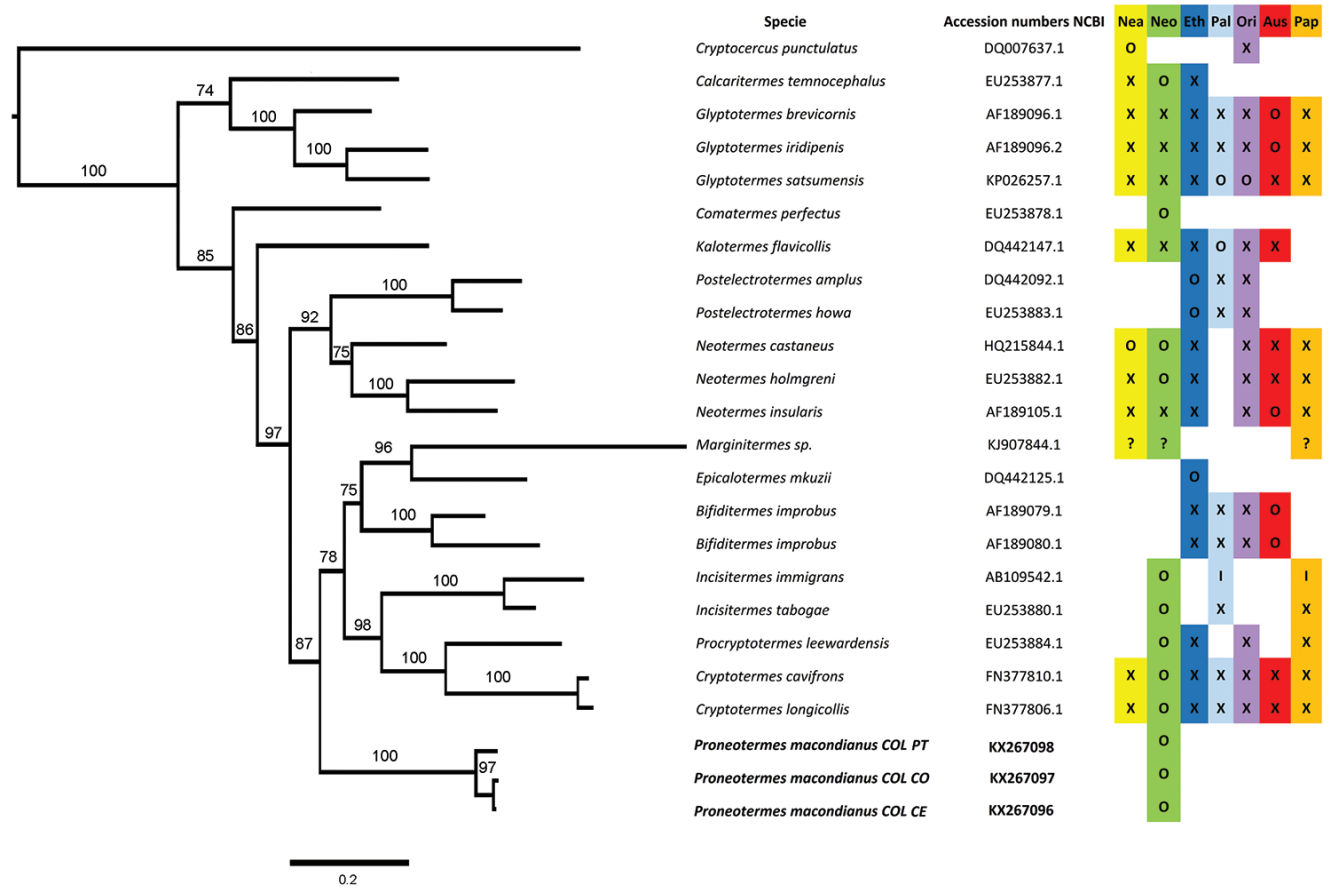
Bayesian inference tree, inferred with MRBAYES from COII sequence data (nodes show posterior probability support). Kalotermitidae distribution (X), Species distribution known (O), unknown (?) and established introductions from other regions (I). Neartic = Nea, Neotropic = Neo, Ethiopian = Eth, Paleartic = Pal, Oriental = Ori, Australian = Aus, Papuan = Pap. Distribution based on Krishna et al. 2013.

The COII, 12S, and 16S sequences obtained in this study are deposited in GenBank under accession numbers KX267090-KX267098 (Table 1). The combined COII, 12S, and 16S nucleotide data of three different haplotypes of *Proneotermes macondianus* from the northern Colombian coast (separated ~200 km from each other) revealed genetic distances of about 2.5% (*p*-distance, SE 0.004; 38bp / 1488bp) (Table 4). Our Bayesian phylogenetic tree shows more than 74% Bayesian Posterior Probability (BPP) support for all nodes (Fig. 5). The COII based tree suggests that *Proneotermes* is the sister taxon to a cluster consisting of [(*Marginitermes* + *Epicalotermes*) + *Bifiditermes*] + [(*Cryptotermes* + *Procrypotermes*) + *Incisitermes*]. Since Kalotermitid sequences for 12S and 16S were very sparse in public databases such as The Barcode of Life Data Systems (BOLD) or NCBI, we only used COII to estimate genetic distances between species and for phylogenetic tree inference. Inter-generic COII *p*-distances for available genera (Table 5) showed that *Bifiditermes* is closest to *Proneotermes* (0.153 SE 0.015).

**Table 4.** Nucleotide distances for combined analysis of COII, 12S rDNA and 16S rDNA genes between localities of *Proneotermes macondianus* sp. n. (*p*-distance). Standard error estimates are shown above the diagonal.

No.	Species (n = number of sequenced specimens)	Localities	CE	CO	PT
1	*Proneotermes macondianus* n = 1	CE		0.002	0.004
2	*Proneotermes macondianus* n = 4	CO	0.004		0.004
3	*Proneotermes macondianus* n = 1	PT	0.024	0.025	

Samples localites: CE = El Ceibal (Santa Catalina, Bolívar) , CO = Los Primates (Colosó, Sucre) , PT = Parque Tayrona (Santa Marta, Magdalena)

**Table 5.** Nucleotide distances for COII sequences between sample localities *Proneotermes macondianus* sp. n, the cockroach *Cryptocercus punctulatus* and different Kalotermitidae (*p*-distance). Standard error estimates are shown above the diagonal.

No.	Specie	1	2	3	4	5	6	7	8	9	10	11	12	13	14	15	16
1	Proneotermes macondianus CE* KX267097		0.003	0.008	0.015	0.016	0.016	0.016	0.017	0.017	0.016	0.017	0.016	0.017	0.018	0.017	0.022
2	Proneotermes macondianus CO** KX267098	0.007		0.008	0.015	0.016	0.017	0.016	0.017	0.017	0.016	0.016	0.016	0.017	0.017	0.017	0.022
3	Proneotermes macondianus PT*** KX267096	0.040	0.044		0.016	0.016	0.016	0.016	0.017	0.017	0.017	0.017	0.017	0.017	0.017	0.017	0.021
4	Bifiditermes improbus AF189080.1	0.153	0.157	0.164		0.016	0.015	0.017	0.015	0.016	0.016	0.017	0.016	0.017	0.017	0.016	0.020
5	Postelectrotermes howa EU253883.1	0.176	0.176	0.174	0.159		0.016	0.018	0.015	0.016	0.016	0.019	0.016	0.017	0.018	0.017	0.021
6	Neotermes castaneus HQ215844.1	0.184	0.179	0.177	0.165	0.157		0.017	0.016	0.017	0.017	0.018	0.016	0.018	0.017	0.016	0.022
7	Incisitermes immigrans AB109542.1	0.175	0.179	0.172	0.188	0.205	0.191		0.018	0.017	0.018	0.017	0.017	0.018	0.019	0.018	0.023
8	Epicalotermes mkuzii DQ442125.1	0.182	0.185	0.189	0.160	0.166	0.176	0.189		0.017	0.016	0.018	0.016	0.017	0.017	0.016	0.022
9	Kalotermes flavicollis DQ442147.1	0.185	0.186	0.188	0.192	0.183	0.192	0.200	0.188		0.017	0.018	0.016	0.018	0.017	0.017	0.022
10	Glyptotermes brevicornis AF189096.1	0.187	0.187	0.197	0.185	0.184	0.197	0.214	0.196	0.200		0.018	0.017	0.018	0.016	0.018	0.022
11	Cryptotermes cavifrons FN377810.1	0.186	0.189	0.186	0.182	0.213	0.202	0.190	0.187	0.211	0.224		0.018	0.016	0.018	0.018	0.023
12	Comatermes perfectus EU253878.1	0.196	0.192	0.209	0.176	0.175	0.181	0.212	0.189	0.202	0.191	0.215		0.017	0.017	0.017	0.022
13	Procryptotermes leewardensis EU253884.1	0.199	0.199	0.189	0.194	0.176	0.187	0.189	0.194	0.202	0.219	0.160	0.204		0.018	0.019	0.022
14	Calcaritermes temnocephalus EU253877.1	0.206	0.209	0.196	0.199	0.213	0.192	0.214	0.194	0.203	0.169	0.212	0.199	0.218		0.017	0.023
15	Marginitermes sp. 9MH1 KJ907844.1	0.209	0.211	0.211	0.193	0.185	0.196	0.231	0.185	0.221	0.230	0.219	0.211	0.225	0.216		0.021
16	Cryptocercus punctulatus DQ007637.1	0.282	0.282	0.279	0.254	0.255	0.281	0.291	0.261	0.266	0.267	0.280	0.266	0.279	0.290	0.257	

Samples localites: CE* = El Ceibal (Santa Catalina, Bolívar) , CO** = Los Primates (Colosó, Sucre) , PT*** = Parque Tayrona (Santa Marta, Magdalena)

##### Ecological notes.

**Figure S1.**
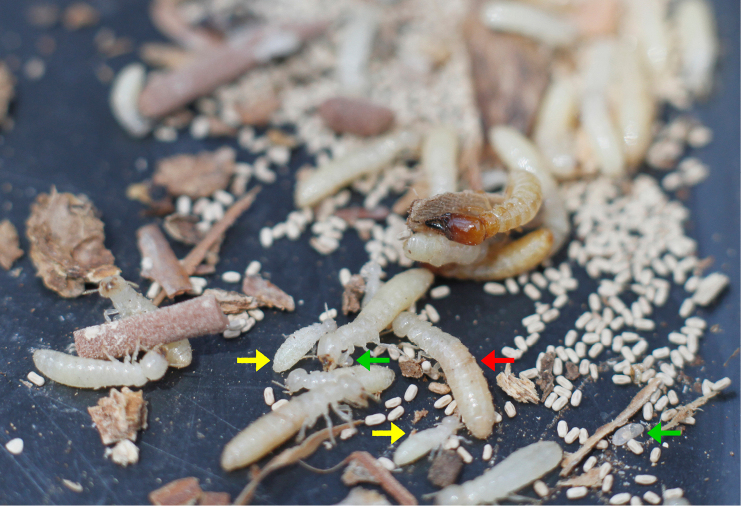
Macrophotography of *Proneotermes macondianus* sp. n. alive from National Park Tayrona. Santa Marta. Magdalena Colombia. Two soldiers (one partly hidden), some pseudergates (whitish, red arrow), two larva (yellow arrow), two eggs (green arrow), and some dry pellets.

**Figure S2.**
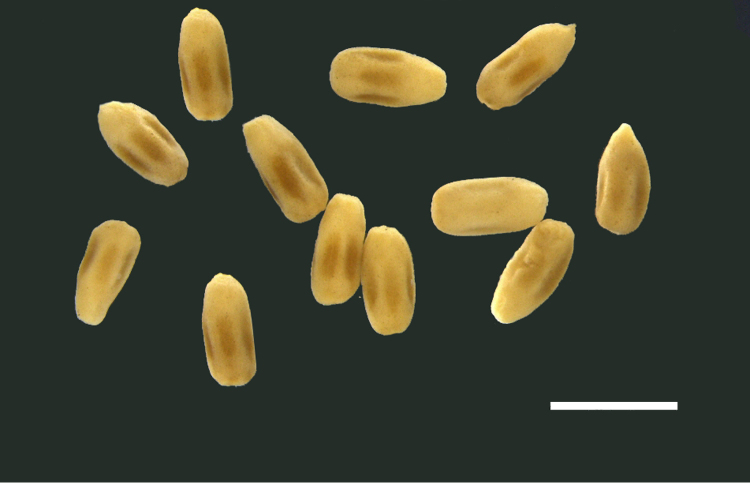
Dry pellets of *Proneotermes macondianus* sp. n. Scale bar 1 mm.

*Proneotermes macondianus* sp. n was found in tropical dry forests of the Colombian Caribbean near to coastal areas up to 25 km inland (Fig. 1, Appendix 1 - Figure S1). Encounters of *Proneotermes* were scarce. In line transects that covered a total area of 1500m × 2m, only 0.82% of all termite samples (n = 1102) were *Proneotermes macondianus* (n = 9). All samples were from thin pieces of drywood branches: less than 2 cm diameter on the ground, with a maximum of 20 individuals per branch. Pellets were hexagonal in shape, beige in colour and had a length of 0.92 +/- 0.04 mm (Appendix 1: Figure S1, S2, Table S1). It was impossible to identify the plant species from the small dry branches where *Proneotermes macondianus* sp. n. was found.

##### Material examined.

Holotype colony: **Colombia**: Municipality of Santa Marta, Magdalena. Tayrona National Natural Park, Gairaca Bay: 11.3152°N, 74.1032°W (Fig. 1), 6m, 27.VI.15 by R. Casalla. COLPT4K1-206. Holotype: Soldier, paratypes: 5 soldiers, 2 reproductives, and few pseudergates, two used for DNA analysis. Municipality of Santa Catalina, Bolívar. Protected area “El Ceibal”: 10.6336°N, 75.2517°W, 25m, 30.VIII.14 R. Casalla. COLCE3F5-155, COLCE3G5-158, COLCE3H2-160: Paratypes: 4 soldiers, 5 functional reproductives, and few pseudergates, two used for DNA analysis. Municipality of Colosó, Sucre. Serranía de Coraza y Montes de María. Protected área “Los Primates”: 9.5332°N, 75.3479°W, 223m, 27.VII.14 R. Casalla. COLCO4F4-226: Paratypes: 1 soldier, 1 winged imago, 2 dealated imagoes, one used for DNA analysis. Measurements for holoype, paratype soldiers and imagoes are reported in Table 2 and 3. The holotype and clearly colored paratype soldiers from COLCE3F5-155 will be deposited in the Arthropod Collection of the Natural History Museum of the Alexander von Humboldt Institute of Bogotá, Colombia (MIAvH). A paratype soldier from holotype colony, will be deposited in the American Museum of Natural History, New York, United States. Morphotype imagoes, paratype soldiers and pseudergates will be part of the collection of the Department of Chemistry and Biology at the University del Norte, Barranquilla, Colombia.

##### Etymology.

*Macondianus*: In honour of Nobel laureate Gabriel García Marquez and the fictional town “Macondo” in his novel “One hundred years of solitude“. “Macondiano/a” is also a Spanish world used in Colombia to describe an incredible, rare or surprising event that could only be compared with the fictional universe and magical realism of this novel.

### Redescription of *Proneotermes latifrons*

Silvestri‘s (1901, 1903) descriptions of the *Proneotermes latifrons* soldier are incomplete. Some characters such as frons angle, horns, and postmentum morphology were not included. Also morphometrical measurements are incomplete. Herein, we included morphometrical measurements for the soldier caste. The imago caste is unknown.

#### Proneotermes latifrons

Taxon classificationAnimaliaIsopteraKalotermitidae

(Silvestri, 1901)

##### Material examined.

**Venezuela**: Bolivar State, El Pauji: 4.4675°N, 61.5947°W, 600m, 25.VII.2003, J Perozo, University of Florida no. SA336: 2 soldiers and pseudergates. Falcon State, La Chapa: 11.2657°N, 69.6022°W, 703m, 27.V.2008, Scheffrahn et al., VZ833, 2 soldiers, pseudergates. Lara State, Copeyal: 10.4409°N, 69.4402°W, 590m, 28.V.2008, Scheffrahn et al., VZ1014, 10 soldiers, pseudergates. Yaracuy State, Licua: 10.3377°N, 69.1344°W, 650m, 30.V.2008, Scheffrahn et al., VZ1180-11183, 4 colonies, many soldiers, pseudergates.

##### Soldier

(Fig. 4D–F, Table 6). Head in dorsal view with frons dark glossy until faint bridge, grading from ferruginous orange to orange-yellow toward vertex. Postclypeus whitish at borders. Mandibles black anteriorly and reddish brown at hump. Head in lateral view with dark ferruginous orange, then turns orange to genal region. Head in ventral view with postmentum chestnut-dark brown and whitish at anterior border and genal margin pale orange. Eye spots distinct, unpigmented. Pronotum hyaline with sclerotized borders. First three antennal segments darker.

**Table 6.** Morphometrical measurements for soldiers of *Proneotermes latifrons*.

No.	Measurements in mm (n = 11).	Mean	SD	Range
1	Head length to tip of mandibles	2.96	0.19	2.65–3.35
2	Head length to frontal horns	1.82	0.31	1.10–2.25
3	Frontal flange width	1.06	0.12	0.90–1.25
4	Genal horns, outside span	1.33	0.12	1.20–1.55
5	Head width max.	1.46	0.12	1.15–1.60
6	Head height excluding postmentum	1.17	0.14	1.00–1.50
7	Pronotum max. width	1.49	0.12	1.20–1.65
8	Pronotum max. length	0.82	0.09	0.70–0.95
9	Left mandible length, tip to ventral condyle	1.06	0.03	1.00–1.10
10	Total length	7.38	0.61	6.80–8.80
11	No. antennal articulations	11	0.7	10–12

Head subsquare with sides slightly convergent, posteriorly and rounded to vertex. Frontal area wide and long, occupies ca 2/5 of head length to postclypeus; narrowly depressed in center, and laterally view with faintly convex and few undulations, sloping angle ca. 50° near to postclypeus. Labrum short and sub-squared. Antennal socket protruded with third antennal segment longer and sclerotized, formula 2<3>4=5=6 and 11 articulations. Postmentum very broad in front. Pronotum as broad as head with anterior emarginated. Mandibles strong and curved inward ca. 45-50°. Measurements are reported in Table 6.

##### Comparisons.

Soldiers of *Proneotermes latifrons* are separated from congeners in having a wide and darker convex frons with narrow undulations dorso-laterally. Postclypeus whitish at border, labrum wider than long and darker postmentum. *Proneotermes latifrons* is distributed in Venezuela, while *Proneotermes perezi* is widely distributed in Central America, from Guatemala to Panama (Fig. 1).

### Additional descriptions for *Proneotermes perezi*

**Material examined. Guatemala**: San Jose La Arada: 14.6965°N, 89.6255°W, 992m, 3.VI.2006, Scheffrahn et al., GUA768-769, 2 colonies many soldiers, pseudergates3 alates. Ipala: 14.5992°N, 89.6411°W, 873m, 3.VI.2006, Scheffrahn et al., GUA793, 5 pseudergates 6 km NW Jutiapa: 14.3307°N, 89.8622°W, 964m, 3.VI.2006, Scheffrahn et et al., GUA822-824, 3 colonies 3 soldiers, many pseudergates, many alates. San José Acatempa: 14.2537°N, 90.1259°W, 1277m 3.VI.2006, Scheffrahn et al., GUA845-851, 7 colonies many soldiers, pseudergates, 4 alates. **Honduras**: P. N. Capiro summit: 15.8697°N, 85.9564°W, 942m, 29.V.2007, Scheffrahn et al., HN217, 5 soldiers, many pseudergates. Amarateca: 14.2247°N, 87.3765°W, 991m, 2.VI.2007, Scheffrahn et et al., HN693, 1 soldier, many pseudergates. **Panama**: Gamboa: 9.12°N, 79.70°W, 9.VI.2005, W. Reeves, University of Florida no. CTA48, 2 alates after rain. Valle de las Minas: 8.6369°N, 82.2114°W, 1050m, Scheffrahn et al., 1.VI.2010, PN1166-1167, 2 colonies many soldiers, pseudergates.

### *Proneotermes perezi* (Holmgren, 1911)

Krishna (1961) described alates and soldiers of *Proneotermes perezi* but did not include measurements. Herein, we report measurements of the soldier, along with new locations and images (Figs 1, 4G–I, Table 7).

**Table 7.** Morphometrical measurements for soldiers of *Proneotermes perezi*.

No.	Measurements in mm (n = 10).	Mean	SD	Range
1	Head length to tip of mandibles	3.36	0.23	3.05–3.80
2	Head length to frontal horns	2.23	0.09	2.10–2.35
3	Frontal flange width	0.93	0.11	0.75–1.10
4	Genal horns outside span	1.41	0.11	1.20–1.60
5	Head width max.	1.52	0.09	1.30–1.65
6	Head height excluding postmentum	1.24	0.08	1.10–1.40
7	Pronotum max. width	1.46	0.13	1.20–1.55
8	Pronotum max. length	0.90	0.08	0.80–1.00
9	Left mandible length, tip to ventral condyle	1.28	0.08	1.15–1.40
10	Total length	7.88	0.60	7.00–8.80
11	No. antennal articulations	12	0.9	11–14

#### Genus Proneotermes

Taxon classificationAnimaliaIsopteraKalotermitidae

Holmgren, 1911

##### Redescription.

Soldiers with head robust, dark coloration in frons, nearly black, grading from ferruginous orange to orange-yellow toward vertex. Frons sloping between 43–50° to postclypeus, without a ridge and vestigial rugosity. Mandibles bended upwards, with a strong dentition and left mandible bigger than right. Mandibular humps pronounced. Eyes spot unpigmented. Third antennal article larger than second and fourth and sclerotized. Pronotum almost as broad as head width. Femur tick, short and strong. Tibal spurs 3:3:3.

Based on our measurements and morphological description, we developed an identification key for the three *Proneotermes* species

### Key to the species *Proneotermes* based on soldier caste

**Table:** 

1	Smaller species; maximum head width 1.14–1.32 mm (mean 1.23 SD 0.06 mm). Lateral margins of the genal horns angle outward from the sides of the head capsule (Fig. 4A, C)	***Proneotermes macondianus* sp. n**
–	Larger species; maximum head width1.15-1.65 mm (mean 1.49 SD 0.11 mm); lateral margins of the genal horns remain in line with the head capsule (Figs 4F, I)	**2**
2	In lateral view; frons forms even curve below vertex and mandibles (Fig. 4E); postmentum about twice as long as wide. Posterior margin conical (Fig. 4F)	***Proneotermes latifrons***
–	In lateral view; frons forms rather straight angle from vertex to mandibles (Fig. 4H); postmentum about three fifths as long as wide. Posterior margin convex (Fig. 4I)	***Proneotermes perezi***

## Discussion

The phylogenetic relationships within the Kalotermitidae are not clearly resolved yet. Krishna (1961) hypothesized that, based on wing venation and morphology of imago mandibles, *Proneotermes* is sister group to a clade composed of *Tauritermes*, *Allotermes*, *Mariginitermes*, and *Incisitermes*. In contrast, our phylogenetic tree shows *Proneotermes*, a Neotropical group, is monophyletic (87% BPP), separated from ancestral line of those who originated *Marginitermes* and the Old World genus *Bifiditermes*, *Epicalotermes*, and New World *Incisitermes* and the Pantropical *Cryptotermes*. However, our results suggest that the genetic distances between *Proneotermes* and congeners are quite high (*p*-distance 0.153 – 0.211 for COII fragment) and the closest genus to *Proneotermes* is *Bifiditermes* (Table 4).

Using a single mitochondrial marker (COII) available for 12 Kalotermitidae genera, our results resemble those from Legendre et al. (2008), who included seven gene fragments in combination with morphological characters in their analyses. To fully resolve phylogenetic relationships within the cosmopolitan Kalotermitidae, a denser taxon sampling along with covering more genetic markers, ideally including nuclear loci and moreover morphological characters is needed.

## Supplementary Material

XML Treatment for Proneotermes macondianus

XML Treatment for Proneotermes latifrons

XML Treatment for Proneotermes

## Appendix 1

**Table S1.** Size of pellet of *Proneotermes macondianus* sp. n.

Pellet	Length (mm)
1	0.92
2	0.86
3	0.92
4	0.91
5	0.96
6	0.96
7	0.91
8	0.97
9	0.95
10	0.87
11	0.9
**Mean**	**0.92**
**SD**	**0.04**
